# Supplementary material for: Introduction of precordial Doppler ultrasound to confirm correct peripheral venous access during general anesthesia in children: A preliminary study
Source: PLoS One. 2021 Mar 22;16(3):e0248999. doi: 10.1371/journal.pone.0248999 (PMC7984609; doi:10.1371/journal.pone.0248999)
Supplement: S1 Table — (DOCX) [file pone.0248999.s001.docx]

S1 Table. Cross-tabulation for each threshold value of change in Doppler flow velocity　(N=512)

1. < 1.0 m/sec

|  | | PIV infiltration and dysfunction | |
| --- | --- | --- | --- |
|  |  | Yes | No |
| Change in Doppler flow velocity | < 1.0 | 4 | 16 |
|  | ≧1.0 | 3 | 489 |

1. < 1.5 m/sec

|  | | PIV infiltration and dysfunction | |
| --- | --- | --- | --- |
|  |  | Yes | No |
| Change in Doppler flow velocity | < 1.5 | 4 | 31 |
|  | ≧1.5 | 3 | 474 |

1. < 2.0 m/sec

|  | | PIV infiltration and dysfunction | |
| --- | --- | --- | --- |
|  |  | Yes | No |
| Change in Doppler flow velocity | < 2.0 | 5 | 52 |
|  | ≧2.0 | 2 | 453 |

1. < 2.5 m/sec

|  | | PIV infiltration and dysfunction | |
| --- | --- | --- | --- |
|  |  | Yes | No |
| Change in Doppler flow velocity | < 2.5 | 5 | 67 |
|  | ≧2.5 | 2 | 438 |

1. < 3.0 m/sec

|  | | PIV infiltration and dysfunction | |
| --- | --- | --- | --- |
|  |  | Yes | No |
| Change in Doppler flow velocity | < 3.0 | 5 | 86 |
|  | ≧3.0 | 2 | 419 |

1. < 3.5 m/sec

|  | | PIV infiltration and dysfunction | |
| --- | --- | --- | --- |
|  |  | Yes | No |
| Change in Doppler flow velocity | < 3.5 | 5 | 100 |
|  | ≧3.5 | 2 | 405 |

1. < 4.0 m/sec

|  | | PIV infiltration and dysfunction | |
| --- | --- | --- | --- |
|  |  | Yes | No |
| Change in Doppler flow velocity | < 4.0 | 5 | 116 |
|  | ≧4.0 | 2 | 389 |

1. < 4.5 m/sec

|  | | PIV infiltration and dysfunction | |
| --- | --- | --- | --- |
|  |  | Yes | No |
| Change in Doppler flow velocity | < 4.5 | 5 | 130 |
|  | ≧4.5 | 2 | 375 |

1. < 5.0 m/sec

|  | | PIV infiltration and dysfunction | |
| --- | --- | --- | --- |
|  |  | Yes | No |
| Change in Doppler flow velocity | < 5.0 | 6 | 146 |
|  | ≧5.0 | 1 | 359 |
